# LncRNA RP11-436H11.5, functioning as a competitive endogenous RNA, upregulates BCL-W expression by sponging miR-335-5p and promotes proliferation and invasion in renal cell carcinoma

**DOI:** 10.1186/s12943-017-0735-3

**Published:** 2017-10-25

**Authors:** Kefeng Wang, Wei Jin, Yan Song, Xiang Fei

**Affiliations:** Department of Urology, Shengjing Hospital of China Medical University, Shenyang, 110004 China

**Keywords:** Non-coding RNAs, RP11-436H11.5, miR-335-5p, Competitive endogenous RNA, Renal cell carcinoma

## Abstract

**Background:**

Accumulating evidence indicates that long non-coding RNAs (lncRNAs) play a crucial role in tumorigenesis. Here, we report a novel lncRNA, RP11-436H11.5, that regulates renal cell carcinoma (RCC) cell proliferation and invasion by sponging miR-335-5p.

**Methods:**

Expression of lncRNA RP11-436H11.5 was determined by a qRT-PCR assay in RCC tissues. RCC cell proliferation and invasion were measured by a cell proliferation assay and a transwell invasion assay. Expression of BCL-W was detected by a western blot assay. Interactions between lncRNA RP11-436H11.5 and miR-335-5p were measured by a luciferase reporter assay and a RNA-pull down assay. In vivo experiments were used to detect tumor formation.

**Results:**

In this study, the qRT-PCR results illustrated that lncRNA RP11-436H11.5 was more highly expressed in RCC tissues than in adjacent normal renal tissues. The results of survival analysis indicated that patients in the high lncRNA RP11-436H11.5 group presented significantly worse outcomes compared with those in the low lncRNA RP11-436H11.5 group. Downregulation of lncRNA RP11-436H11.5 suppressed RCC cell proliferation and invasion in vitro and in vivo. Luciferase reporter assay results demonstrated that lncRNA RP11-436H11.5 enhanced BCL-W expression by regulating miR-335-5p expression. LncRNA RP11-436H11.5 could function as a miR-335-5p decoy to derepress expression of BCL-W.

**Conclusions:**

LncRNA RP11-436H11.5 could function as a competing endogenous RNA to promote RCC cell proliferation and invasion, which might serve as a therapeutic application to suppress RCC progression.

**Electronic supplementary material:**

The online version of this article (10.1186/s12943-017-0735-3) contains supplementary material, which is available to authorized users.

## Background

Renal cell carcinoma (RCC) is the sixth most common malignant tumor in the United States, representing approximately 5% of adult male malignancies in 2017 [1]. The mortality of RCC patients appears to be increasing each year, resulting in frequent studies on biological detections and treatments [2]. Drug targeted therapies, including mammalian targets of rapamycin and vascular endothelial growth factor, have boomed [3, 4]. Unfortunately, drug resistance can occur in late stage patients, resulting in a bad prognosis [5, 6]. Therefore, the molecular mechanisms of RCC tumorigenesis need to be thoroughly investigated.

Long non-coding RNAs (lncRNAs) are a set of non-protein coding transcripts longer than 200 nucleotides [7]. They have been shown to have various functions including post-transcriptional regulation, chromatin modification and other biological processes [8, 9]. Some lncRNAs have been shown to play crucial roles in different kinds of cancer cells, including breast [10], colorectal [11], gastric [12] and renal [13] cancer cells.

microRNAs (miRNAs) are also non-coding RNAs (ncRNAs) that are well known to play important roles in tumor biological processes [14, 15]. Effective diagnostic and therapeutic strategies have been used in clinical settings worldwide. Recently, a new mechanism was discovered in which some lncRNAs and mRNAs could interact with each other by competing with some common miRNAs [16]. In this case, lncRNAs could function as competing endogenous RNA (ceRNA) to sponge related microRNAs for the derepression of downstream genes at a post-transcriptional level [17, 18]. This mechanism provides a new way to study ncRNAs in tumors.

Our previous study indicated that miR-335 could function as a tumor suppressor in RCC. Expression of miR-335 was downregulated in RCC tissues compared to corresponding normal renal tissues. Low expression of miR-335 was associated with tumor size, lymph node metastasis and T stage. miR-335 could inhibit proliferation and invasion through direct suppression of BCL-W [19]. Whether lncRNAs could regulate miR-335 to influence the biological behaviors of RCC cells has not been characterized.

Here, we first identified a novel lncRNA, RP11-436H11.5, that was more highly expressed in RCC tissues than in paired normal renal tissues. Downregulation of lncRNA RP11-436H11.5 could result in significant suppression of proliferation and invasion in vitro and in vivo. Our data demonstrated that lncRNA RP11-436H11.5 could directly bind with miR-335-5p and function as a miRNA decoy to regulate BCL-W expression.

## Methods

### Clinical samples

Human RCC tissues and adjacent normal renal tissues were acquired from patients diagnosed with RCC in the Department of Urology, Shengjing Hospital of China Medical University. The ethics consents were signed by each patient before the study. All patients agreed that the data from their samples could be used for experimental studies and paper presentations.

### Reagents

Bcl2L2 (BCL-W) antibody was purchased from Abcam Public Limited Company (Product code ab38,629). GAPDH antibody (0411) was from Santa Cruz Biotechnology (Catalog# sc-47,724). All the antibodies were stored at −20°C.

### Cell culture

RCC cells (A498, 786-O and OSRC-2) were purchased from American Type Culture Collection (ATCC, Manassas, VA) and cultured in Dulbecco’s Modified Eagle’s Medium (DMEM, Invitrogen, Grand Island, NY, USA) supplemented with 10% fetal bovine serum (FBS), 1% L-glutamine, penicillin (25 units/ml) and streptomycin (25 g/ml). All cell lines were detected and authenticated as bacteria and mycoplasma free following ATCC’s instructions within the past 3 months.

### Lentivirus packaging

The siRNA sequences targeting lncRNA RP11-436H11.5 were F-primer TAATTTGTTTCTAGATGTGTG and R-primer CACATCTAGAAACAAATTAAT. A Gibson assembly assay was performed for oelncRNA RP11-436H11.5 with F-primer TTTCGACATTTAAATTTAATGCTGTTTTACTTGCACGCAC and R-primer ATTCCTGCAGCCCGTAGTTTCCTACACAAAAACTTGGGTA. miR-335 mimics were synthesized by RiboBio (Guangzhou, Guangdong, China). These plasmids and the psPAX2 packaging plasmid, pMD2G envelope plasmid were transfected into HEK293T cells to get the lentivirus soups following the manufacturer’s protocol and frozen in −80°C for use.

### RNA extraction and qRT-PCR assay

Total RNAs were extracted using Trizol reagent (Invitrogen, Grand Island, NY). RNAs were reverse transcribed by Superscript III transcriptase (Invitrogen, Grand Island, NY). QRT-PCR was applied using a Bio-Rad CFX96 system with SYBR green to detect the mRNA expression level of a gene of interest. The qRT-PCR protocol was as follows: 50°C for 2 min, 95°C for 8 min 30 s, followed by 45 cycles at 95°C for 15 s, and 60°C for 1 min. The extension is 95°C for 1 min, 55°C for 1 min, and 55°C for 10 s. GAPDH was used as a normalized control.

miRNAs were extracted using a PureLink® miRNA kit. The qRT-PCR protocol was as follows: 95°C for 2 min, followed by 45 cycles at 95°C for 15 s, and 60°C for 45 s. U6 and/or RPL32 was used as a normalized control.

### Cell proliferation assay

RCC cells were seeded in 24-well plates (3000 cells/well) and cultured for indicated periods. Then media were replaced with MTT regent and DMSO was used to dissolve the blue crystals. Cells proliferation and viability were measured with absorbance at 570 nm.

### Cell invasion assay

RCC cells were seeded in a 6-well plate after designated treatments and incubated for 72 h. The upper chambers were coated with Matrigel (1:20, BD Corning) 2 h before plating the cells. Cells were collected with serum-free media and plated into the upper chambers at a concentration of 1 × 10^5^/ml. A total of 750 μl of 10% FCS media was added into the lower chambers for incubation at 37°C in 5% (*v*/v) CO_2_ incubator for 12–14 h. The invaded cells were permeabilized by methanol for 20 min at room temperature and stained with 0.1% (*w*/*v*) crystal violet in a dark room.

### Luciferase reporter assay

LncRNA RP11-436H11.5 involving wild-type or mutant miRNA response elements (MREs) were cloned into the psiCHECK2 vector (Promega, Madison, WI) at downstream of the Renilla luciferase ORF. A498 and 786-O cells were plated in 24-well plates, and transfected with constructed plasmids with lipofectamine 3000 transfection reagent (Invitrogen, Carlsbad, CA). Luciferase activities were measured 36–48 h after transfection by Dual-Luciferase Assay (Promega) according to the manufacturer’s manual.

### Western blot assay

Protein was extracted with lysis buffer and electrophoretically transferred onto PVDF membranes (Millipore, Billerica, MA). Then the membranes were blocked by Bovine Serum Albumin (Sigma-Aldrich, St. Louis, MO) and bred with primary antibodies at 4°C overnight. Thereafter, the membranes were incubated with secondary antibodies at room temperature for 1 h. Bands were visualized by an ECL chemiluminescent detection system (Thermo Fisher Scientific, Rochester, NY).

### RNA pull-down assay

Cells were quantitated and treated with 1 ml of cell lysis buffer for 72 h. Then, cells were rotated overnight at 4°C after adding 1.5 μl of RNase inhibitor, 10 μl of streptavidin agarose beads and 500 pM antisense oligos. Beads were washed 5 times by cell lysis buffer. Total RNAs were subjected to qRT-PCR analysis.

### Ago2 immunoprecipitation assay

Transfected cells were lysed with RIPA lysis buffer (150 mM NaCl, 20 nM Tris-HCl (PH 7.5), 1% NP-40, 2.5 mM sodium pyrophosphate, 1 mM Na2EDTA, 1 mM EGTA, 1% sodium deoxycholate, 1 μg/ml leupetin, 1 mM Na3VO4, and 1 mM beta-glycerophosphate). Cell suspension was centrifuged 15 min at 14000 rpm. The supernatant was rotated overnight at 4 °C after adding 2 μl of AGO2 antibody and 10 μl of beads. The mixture was washed 3 times with lysis buffer. RNAs were extracted using Trizol reagent (Invitrogen).

### In vivo studies

Twenty-four 6–8-week-old nude mice were purchased from Shanghai SLAC Laboratory Animal Co. Ltd. A498 cells were engineered to express luciferase reporter gene (PCDNA3.0-luciferase) and then stably transfected with pLVTHM, shlncRNA RP11-436H11.5 and oelncRNA RP11-436H11.5. Approximately 1 × 10^6^ A498 cells (mixed with Matrigel, 1:1) were injected into the subrenal capsule. Tumor formation and metastasis were monitored by Fluorescent Imager (IVIS Spectrum, Caliper Life Sciences, Hopkinton, MA) once a week. Mice were sacrificed after 6 weeks. Tumors were removed for study.

### Statistical analysis

Data were expressed as the mean ± SEM from at least 3 independent experiments. Statistical analyses involved paired t-tests with SPSS 17.0 (SPSS Inc., Chicago, IL). Overall survival (OS) was evaluated by Kaplan-Meier survival curves and compared by log-rank test. *P* < 0.05 was considered statistically significant.

## Results

### LncRNA RP11-436H11.5 expression is upregulated in RCC tissues

Recently, many studies have indicated that lncRNAs can regulate gene expression by complementary binding with miRNAs [9, 20]. Our previous study demonstrated that miR-335 suppressed RCC cell proliferation and invasion [19]. We hypothesized that there were some lncRNAs that could regulate RCC cell progression by binding with miR-335. Therefore, we used a human lncRNA target prediction tool (DIANA TOOLS) to predict the potential lncRNAs that can interact with miR-335-5p. We selected the top 14 potential lncRNAs for the next screening strategy (Additional file 1: Figure S1).

**Fig. 1:**
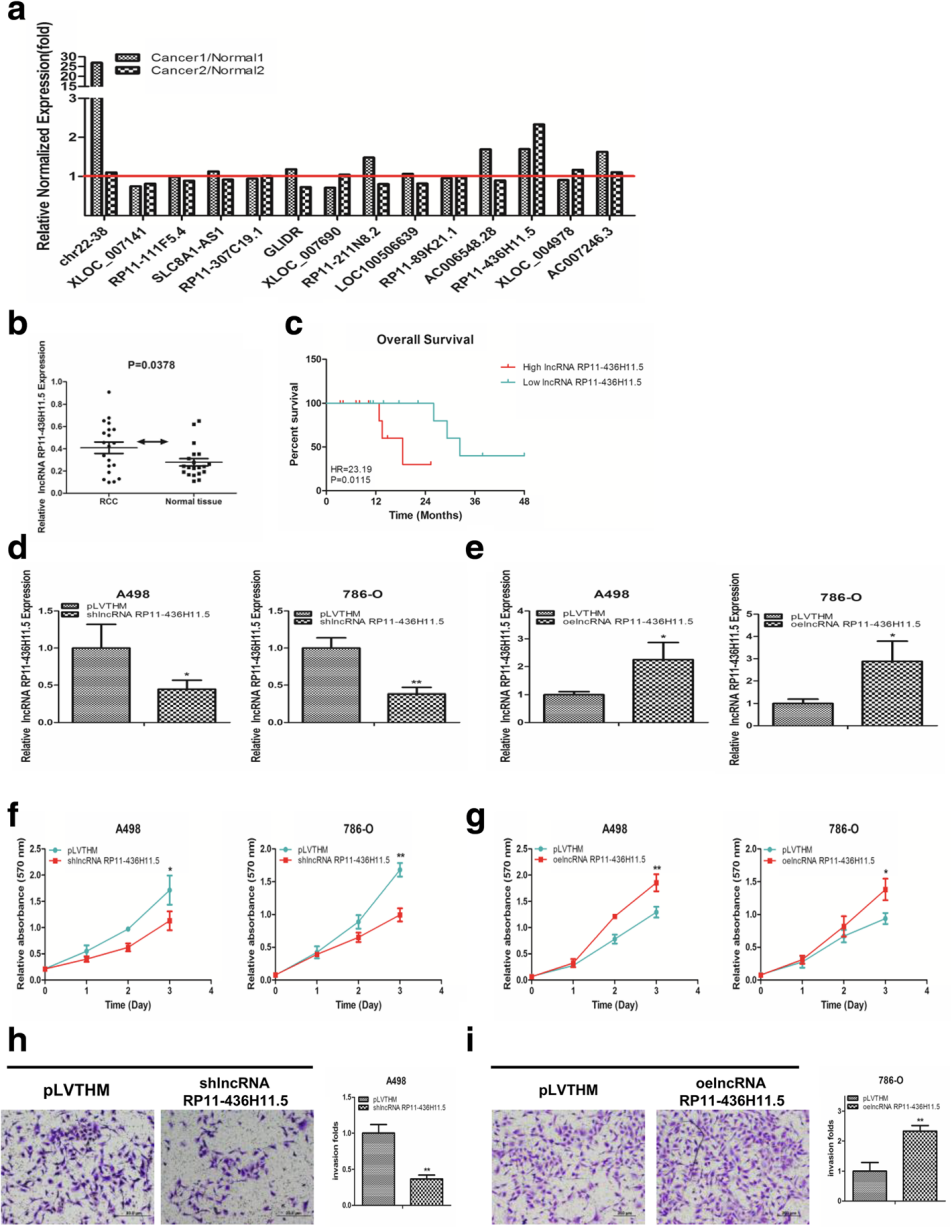
LncRNA RP11-436H11.5 expression is upregulated in RCC tissues and promotes RCC cell proliferation and invasion. **a.** A qRT-PCR assay was performed to screen 14 potential lncRNAs. LncRNA RP11-436H11.5 was the most appropriate lncRNA to select. Each column is the ratio of caner tissue / normal tissue. **b.** LncRNA RP11-436H11.5 expression in RCC tissues vs normal renal tissues. **c.** Association of lncRNA RP11-436H11.5 expression with OS analysis of 20 RCC patients. *P* values are given on the figures. **d** and **e.** Verification of lncRNA RP11-436H11.5 knockdown (**d**) and overexpression (**e**) by qRT-PCR assay. **f** and **g.** A498 and 786-O cells were transfected with lncRNA RP11-436H11.5-shRNA (**f**) and functional lncRNA RP11-436H11.5-cDNA (**g**). Cell growth was measured by a cell proliferation assay. **h** and **i.** A chamber-transwell invasion assay was performed using A498 (**h.** pLVTHM vs shlncRNA RP11-436H11.5) and 786-O (**i.** pLVTHM vs oelncRNA RP11-436H11.5) cells. The invaded cells were counted in 10 randomly chosen microscopic fields (100×) of each experiment and pooled. **d, e, f, g, h** and **i.** Each sample was run in triplicate and in multiple experiments for mean ± SEM. *P < 0.05, **P < 0.01 compared to controls

First, qRT-PCR was performed to detect the expression level of these 14 lncRNAs in 2 matched RCC tissues and adjacent normal renal tissues. The results showed that lncRNA RP11-436H11.5 was the most appropriate lncRNA as it was more highly expressed in RCC tissues than in adjacent normal renal tissues (Fig. 1a). Furthermore, we detected lncRNA RP11-436H11.5 expression in 20 matched primary RCC tissues and adjacent normal renal tissues. The results confirmed that the level of lncRNA RP11-436H11.5 was higher in RCC tissues than in adjacent normal renal tissues (Fig. 1b). Meanwhile, a 4-year OS was analyzed by Kaplan-Meier survival curves. Our data showed that RCC patients in the high lncRNA RP11-436H11.5 group presented significantly worse outcomes compared with those in the low lncRNA RP11-436H11.5 group (Fig. 1c).

### LncRNA RP11-436H11.5 promotes RCC cell proliferation and invasion.

Next, we explored the functional effects of lncRNA RP11-436H11.5 on RCC cells. A498 and 786-O cells were transfected with lncRNA RP11-436H11.5-shRNA and functional lncRNA RP11-436H11.5-cDNA. The qRT-PCR results confirmed that the expression of lncRNA RP11-436H11.5 was effectively modulated in A498 and 786-O cells (Fig. 1d and e).

Functionally, the cell proliferation assay demonstrated that knockdown of lncRNA RP11-436H11.5 significantly decreased cell proliferation, whereas overexpression of lncRNA RP11-436H11.5 significantly increased cell proliferation in A498 and 786-O cells (Fig. 1f and g).

As expected, using a transwell invasion assay, we obtained similar results in both A498 (Fig. 1h) and 786-O cells (Fig. 1i).

Together, these results demonstrated that lncRNA RP11-436H11.5 was more highly expressed in RCC tissues than in adjacent normal renal tissues and significantly promoted RCC cell proliferation and invasion.

### LncRNA RP11-436H11.5 enhances RCC cell progression by upregulating BCL-W expression

Our previous study demonstrated that miR-335-5p could inhibit RCC cell proliferation and invasion through direct suppression of BCL-W [19]. We know that lncRNA RP11-436H11.5 can regulate many downstream genes to influence RCC cell biological behaviors. We attempted to explore whether BCL-W is one of the downstream genes of lncRNA RP11-436H11.5.

**Fig. 2:**
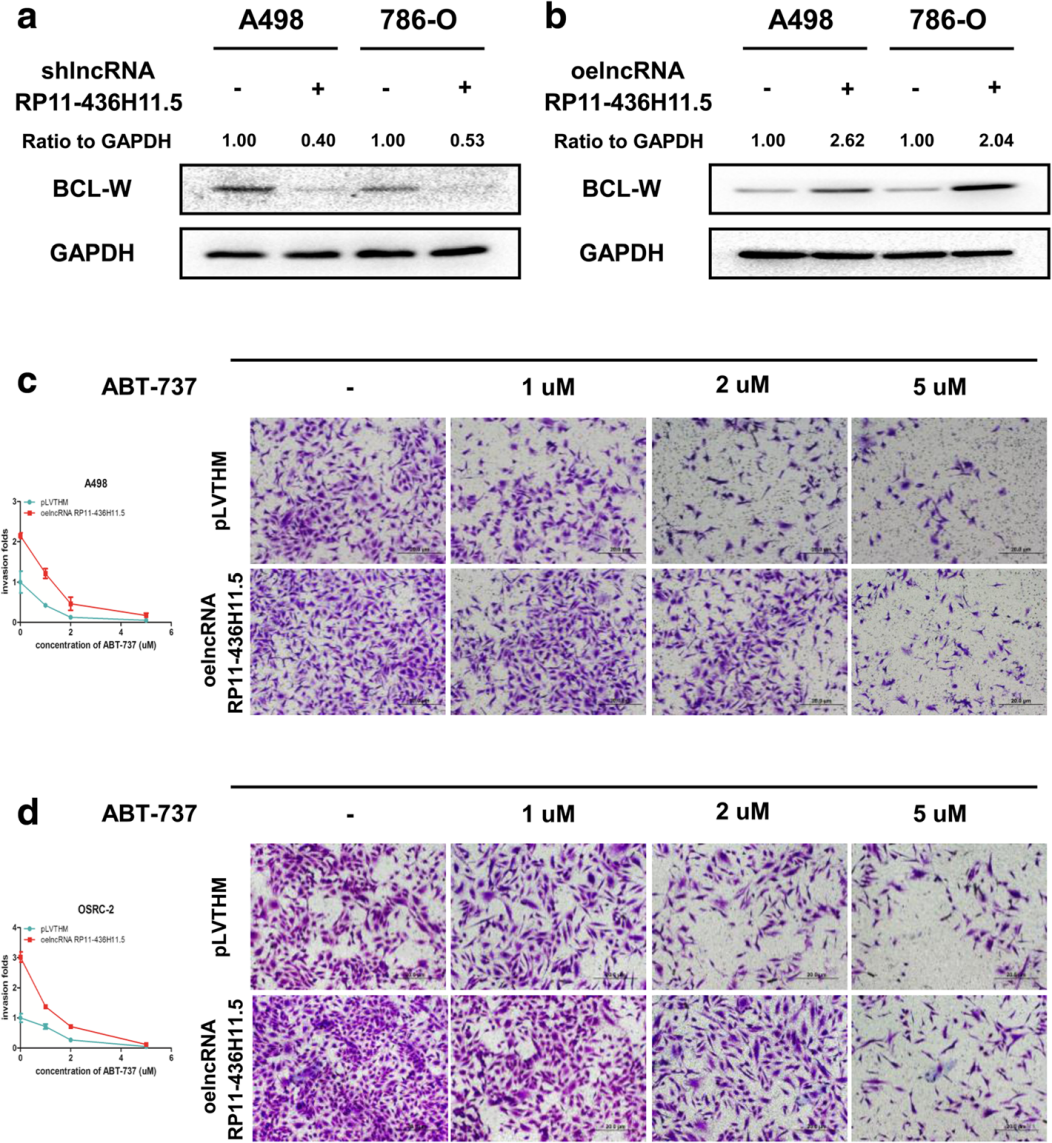
LncRNA RP11-436H11.5 enhances RCC cell progression by upregulating BCL-W expression. **a** and **b.** A498 and 786-O cells were transfected with lncRNA RP11-436H11.5-shRNA (**a**) and functional lncRNA RP11-436H11.5-cDNA (**b**) separately. The expression of BCL-W (ratio to GAPDH) was measured by western blot assay. **c** and **d.** A chamber-transwell invasion assay was performed using A498 (**c**) and OSRC-2 (**d**) cells transfected with functional lncRNA RP11-436H11.5-cDNA, control and different concentrations of ABT-737

As our expected, western blot assay revealed that knocking down lncRNA RP11-436H11.5 decreased the expression of BCL-W compared to the control in both A498 and 786-O cells (Fig. 2a). In contrast, overexpression of lncRNA RP11-436H11.5 increased BCL-W expression (Fig. 2b).

Furthermore, we investigated the effect of ABT-737, a small molecule inhibitor of BCL-W, on lncRNA RP11-436H11.5-mediated RCC cell invasion. The results showed that ABT-737 dramatically reversed lncRNA RP11-436H11.5-increased RCC cell invasion in A498 and OSRC-2 cells in a concentration-dependent way (Fig. 2c and d).

Together, the results from Fig. 2a-d showed that lncRNA RP11-436H11.5 upregulated BCL-W expression to promote RCC cell progression.

### LncRNA RP11-436H11.5 regulates BCL-W expression via miR-335-5p

Since lncRNA RP11-436H11.5 was positively associated with RCC cell progression, we next investigated how lncRNA RP11-436H11.5 regulated BCL-W expression in RCC cell proliferation and invasion.

**Fig. 3:**
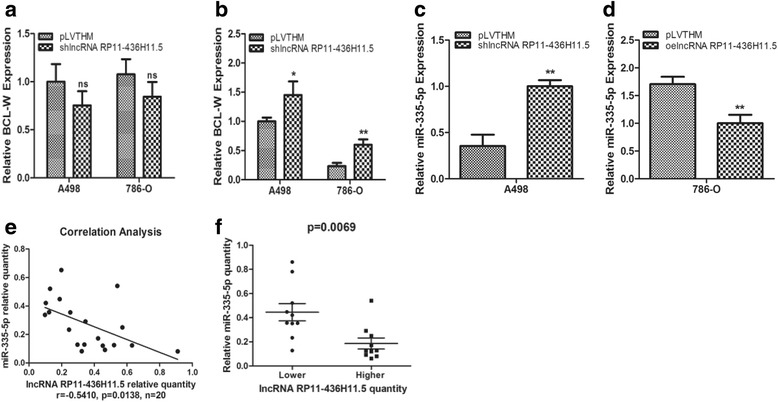
LncRNA RP11-436H11.5 regulates BCL-W expression via miR-335-5p. **a.** A qRT-PCR assay was performed to detect the expression of BCL-W after knocking down lncRNA RP11-436H11.5 in A498 and 786-O cells. **b.** An argonaute2 immunoprecipitation assay was performed after transfecting with lncRNA RP11-436H11.5-shRNA in A498 and 786-O cells. **c** and **d.** A qRT-PCR assay was performed to detect the expression of miR-335-5p after knocking down lncRNA RP11-436H11.5 in A498 cells (**c**) and overexpressing lncRNA RP11-436H11.5 in 786-O cells (**d**). **e.** Correlation analysis of miR-335-5p and lncRNA RP11-436H11.5 by Pearson’s correlation coefficient. **f.** miR-335-5p level was measured in patients with low lncRNA RP11-436H11.5 (*n* = 10) and high lncRNA RP11-436H11.5 (n = 10) expression. **a, b, c** and **d.** Each sample was run in triplicate and in multiple experiments for mean ± SEM. *P < 0.05, **P < 0.01 compared to controls

We added lncRNA RP11-436H11.5-shRNA in A498 and 786-O cells and detected BCL-W expression. The qRT-PCR results revealed that the mRNA level of BCL-W changed little compared to the control (Fig. 3a), which implied that lncRNA RP11-436H11.5 might not regulate BCL-W expression directly. Therefore, we considered that some non-coding RNAs might play important roles at a post-transcriptional level in this process. To certify our hypothesis, we performed an Argo2 immunoprecipitation assay and detected the mRNA level of BCL-W. The results revealed that the mRNA level of BCL-W increased after knocking down lncRNA RP11-436H11.5 in A498 and 786-O cells (Fig. 3b), which verified our viewpoints that some miRNAs could participate in a RNA-induced silencing complex responsible for repressing BCL-W mRNA degradation and translation.

Using a human miRNA target tool (DIANA TOOLS), we predicted that lncRNA RP11-436H11.5 might be targeted by miR-335-5p. The predicted binding sites of miR-335-5p to lncRNA RP11-436H11.5 were illustrated in Additional file 2: Figure S2. Therefore, we targeted miR-335-5p for further exploration. As shown on Fig. 3c, the expression of miR-335-5p increased after knocking down lncRNA RP11-436H11.5 in A498 cells. In contrast, overexpression of lncRNA RP11-436H11.5 decreased the expression of miR-335-5p in 786-O cells (Fig. 3d). Importantly, there was an inverse correlation (*r* = −0.5410, *p* = 0.0138) between lncRNA RP11-436H11.5 level and miR-335-5p level in 20 RCC tissues (Fig. 3e), showing that a lower lncRNA RP11-436H11.5 level was associated with a higher miR-335-5p level (Fig. 3f).

Our previous study demonstrated that miR-335-5p could regulate BCL-W expression by binding to the 3′-untranslated region (3′-UTR) of BCL-W mRNA using a luciferase reporter assay [19].

Together, the results from Fig. 3a-f and Additional file 2: Figure S2 demonstrated that lncRNA RP11-436H11.5 could regulate BCL-W expression via miR-335-5p by binding to the 3′-UTR of BCL-W mRNA.

### LncRNA RP11-436H11.5 functions by altering miR-335-5p-BCL-W signals to promote RCC cell proliferation and invasion

**Fig. 4:**
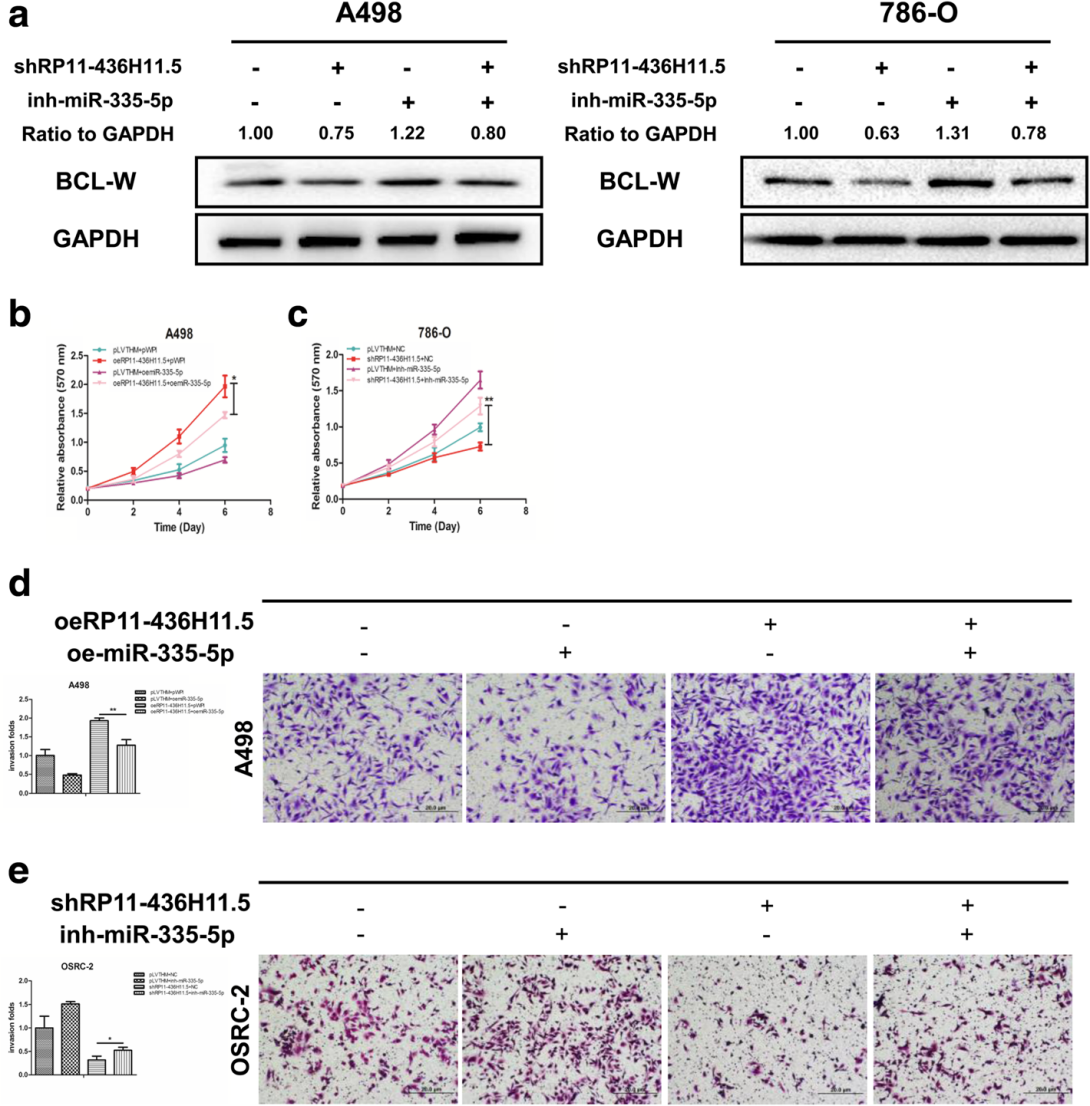
LncRNA RP11-436H11.5 functions by altering miR-335-5p-BCL-W signals to promote RCC cell proliferation and invasion. **a.** Protein level of BCL-W (ratio to GAPDH) was determined by immunoblot analysis after cotransfection with lncRNA RP11-436H11.5-shRNA and miR-335-5p inhibitor. **b.** Adding oemiR-335-5p partially rescued the growth of A498 cells transfected with oelncRNA RP11-436H11.5 by a cell proliferation assay. **c.** Adding miR-335-5p inhibitor partially reversed the growth of 786-O cells transfected with shlncRNA RP11-436H11.5 by a cell proliferation assay. **d.** A chamber-transwell invasion assay was performed using A498 cells with four groups (control, oelncRNA RP11-436H11.5, oemiR-335-5p, and oelncRNA RP11-436H11.5 + oemiR-335-5p). The invaded cells were counted in 10 randomly chosen microscopic fields (100×) of each experiment and pooled. **e.** A chamber-transwell invasion assay was performed using OSRC-2 cells with four groups (control, shlncRNA RP11-436H11.5, miR-335-5p inhibitor, and shlncRNA RP11-436H11.5 + miR-335-5p inhibitor). The invaded cells were counted in 10 randomly chosen microscopic fields (100×) of each experiment and pooled. **b, c, d** and **e.** Each sample was run in triplicate and in multiple experiments for mean ± SEM. *P < 0.05, **P < 0.01 compared to controls

Our previous study demonstrated that miR-335-5p inhibited RCC cell progression through the direct suppression of BCL-W. Therefore, we examined whether lncRNA RP11-436H11.5 could affect RCC cell proliferation and invasion by altering miR-335-5p-BCL-W signals. Western blot assay revealed that knocking down lncRNA RP11-436H11.5 decreased the expression of BCL-W in both A498 and 786-O cells, and this shlncRNA RP11-436H11.5-suppressed BCL-W expression was partially reversed by adding a miR-335-5p inhibitor (Fig. 4a). The results also showed that overexpression of miR-335-5p partially reversed oelncRNA RP11-436H11.5-enhanced RCC cell proliferation in A498 cells (Fig. 4b). On the other hand, miR-335-5p inhibitor partially reversed RCC cell proliferation induced by shlncRNA RP11-436H11.5 in 786-O cells (Fig. 4c).

As expected, we obtained similar results in both A498 (Fig. 4d) and OSRC-2 cells (Fig. 4e) using a transwell invasion assay.

Together, the results from Fig. 4a-e illustrated that lncRNA RP11-436H11.5 could promote RCC cell proliferation and invasion by altering miR-335-5p-BCL-W signals**.**

### LncRNA RP11-436H11.5 works as a miR-335-5p decoy to regulate BCL-W expression in RCC cells

**Fig. 5:**
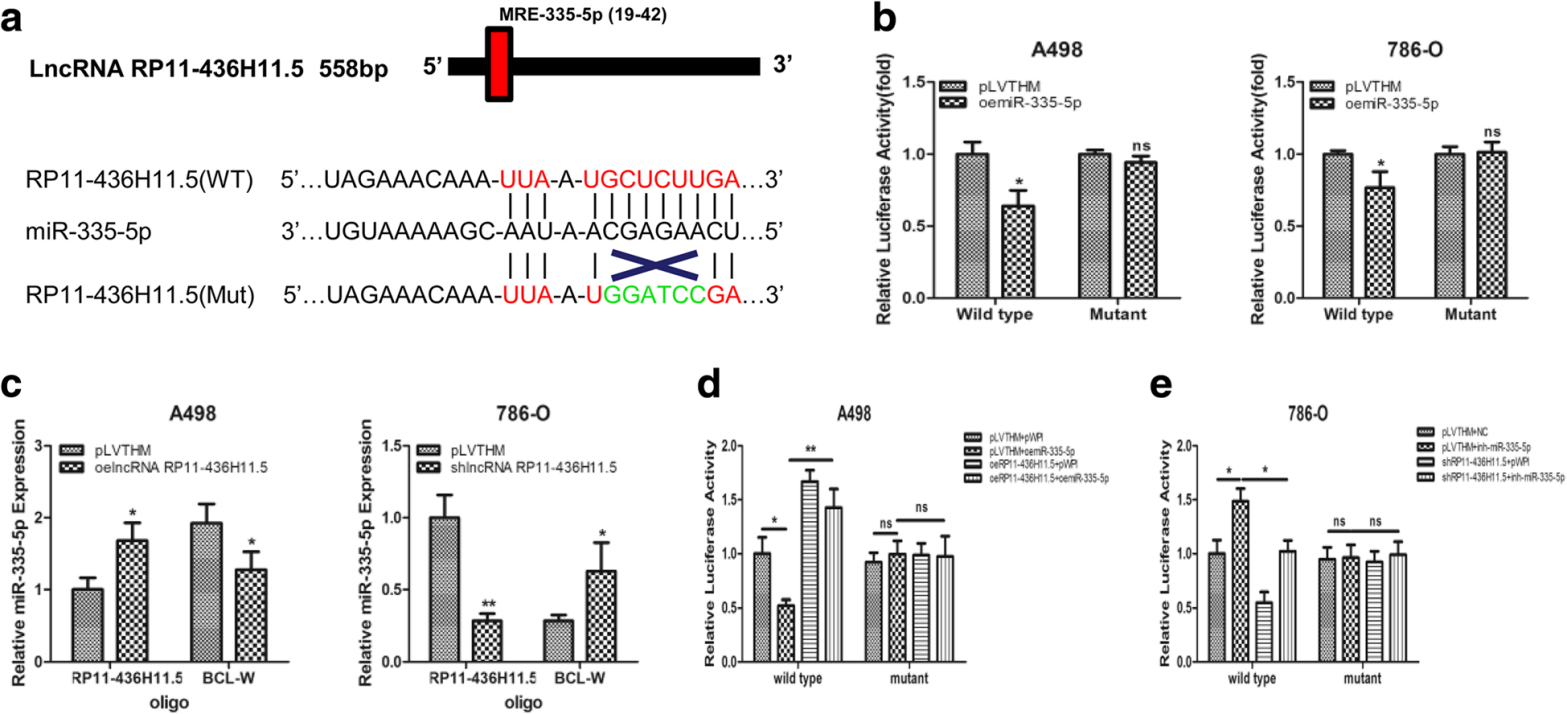
LncRNA RP11-436H11.5 works as a miR-335-5p decoy to regulate BCL-W expression in RCC cells. **a.** Sequence alignment of lncRNA RP11-436H11.5 3′-UTR with wild-type (WT) versus mutant potential miR-335-5p targeting sites. **b.** Cotransfection of wild-type or mutant seed regions of lncRNA RP11-436H11.5 3′-UTR constructed with oemiR-335-5p in A498 and 786-O cells. Luciferase reporter assay was applied to detect the luciferase activities. **c.** A RNA-pull down assay was performed by biotinylated antisense oligo specific to lncRNA RP11-436H11.5 and BCL-W after transfecting with oelncRNA RP11-436H11.5 or shlncRNA RP11-436H11.5 in A498 and 786-O cells. **d.** Cotransfection of wild-type or mutant seed regions of BCL-W 3′-UTR constructed with oemiR-335-5p and oelncRNA RP11-436H11.5 in A498 cells. Luciferase reporter assay was applied to detect luciferase activities. **e.** Cotransfection of wild-type or mutant seed regions of BCL-W 3′-UTR constructed with miR-335-5p inhibitor and shlncRNA RP11-436H11.5 in 786-O cells. Luciferase reporter assay was applied to detect the luciferase activities. **b, c, d** and **e.** Each sample was run in triplicate and in multiple experiments for mean ± SEM. *P < 0.05, **P < 0.01 compared to controls

Over the years, ceRNA appeared to be a vital mechanism for lncRNA and miRNA interaction. To further elucidate whether lncRNA RP11-436H11.5 can function as a ceRNA, we detected the specific binding sites between miR-335-5p and lncRNA RP11-436H11.5. We cloned full-length lncRNA RP11-436H11.5 containing presumptive miR-335-5p binding sites and conducted a luciferase reporter assay (Fig. 5a). The results showed that oemiR-335-5p inhibited the luciferase activities of wild-type binding sites, but failed to do so with mutation of the miR-335-5p binding seed region in lncRNA RP11-436H11.5 in A498 and 786-O cells (Fig. 5b).

Subsequently, a RNA-pull down assay was applied to confirm that miR-335-5p could interact with lncRNA RP11-436H11.5 and BCL-W. Functional lncRNA RP11-436H11.5-cDNA and lncRNA RP11-436H11.5-shRNA were added in 786-O and A498 cells, respectively. Specific biotinylated antisense oligoes were used to isolate lncRNA RP11-436H11.5 and BCL-W from the above RCC cells. The qRT-PCR results revealed that lncRNA RP11-436H11.5 absorbed more miR-335-5p, whereas BCL-W absorbed less miR-335-5p after overexpression of lncRNA RP11-436H11.5 in A498 cells (Fig. 5c). Similar results were obtained when we knocked down lncRNA RP11-436H11.5 in 786-O cells (Fig. 5c).

Finally, luciferase reporter assay was applied through psiCheck2 vector holding wild-type or mutant 3′-UTR of BCL-W. The results showed that luciferase activities decreased after adding oemiR-335-5p in A498 cells (Fig. 5d, pLVTHM + oemiR-335-5p vs pLVTHM + pWPI). The restraint effect of oemiR-335-5p on BCL-W was reversed with the addition of functional lncRNA RP11-436H11.5-cDNA (Fig. 5d, oeRP11-436H11.5 + oemiR-335-5p vs pLVTHM + oemiR-335-5p). No prominent differences were found for mutant 3′-UTR of BCL-W (Fig. 5d). On the other hand, the luciferase activities increased after adding miR-335-5p inhibitor in 786-O cells (Fig. 5e, pLVTHM + inh-miR-335-5p vs pLVTHM + pWPI). The enhanced effect of miR-335-5p inhibitor on BCL-W was reversed with the addition of lncRNA RP11-436H11.5-shRNA (Fig. 5e, shlncRNA RP11-436H11.5 + inh-miR-335-5p vs pLVTHM + inh-miR-335-5p). No prominent differences were found for mutant 3′-UTR of BCL-W (Fig. 5e).

Together, the results from Fig. 5a-e illustrated that lncRNA RP11-436H11.5 could function as a miRNA decoy to defend BCL-W from degradation by miR-335-5p in RCC cells.

### Deregulation of lncRNA RP11-436H11.5 suppresses RCC cell proliferation and invasion in RCC orthotopic xenografts

The above in vitro data indicated that lncRNA RP11-436H11.5 promoted RCC cell proliferation and invasion. Furthermore, orthotopic xenograft mouse models were applied to detect the anti-tumorigenic role of shlncRNA RP11-436H11.5.

**Fig. 6:**
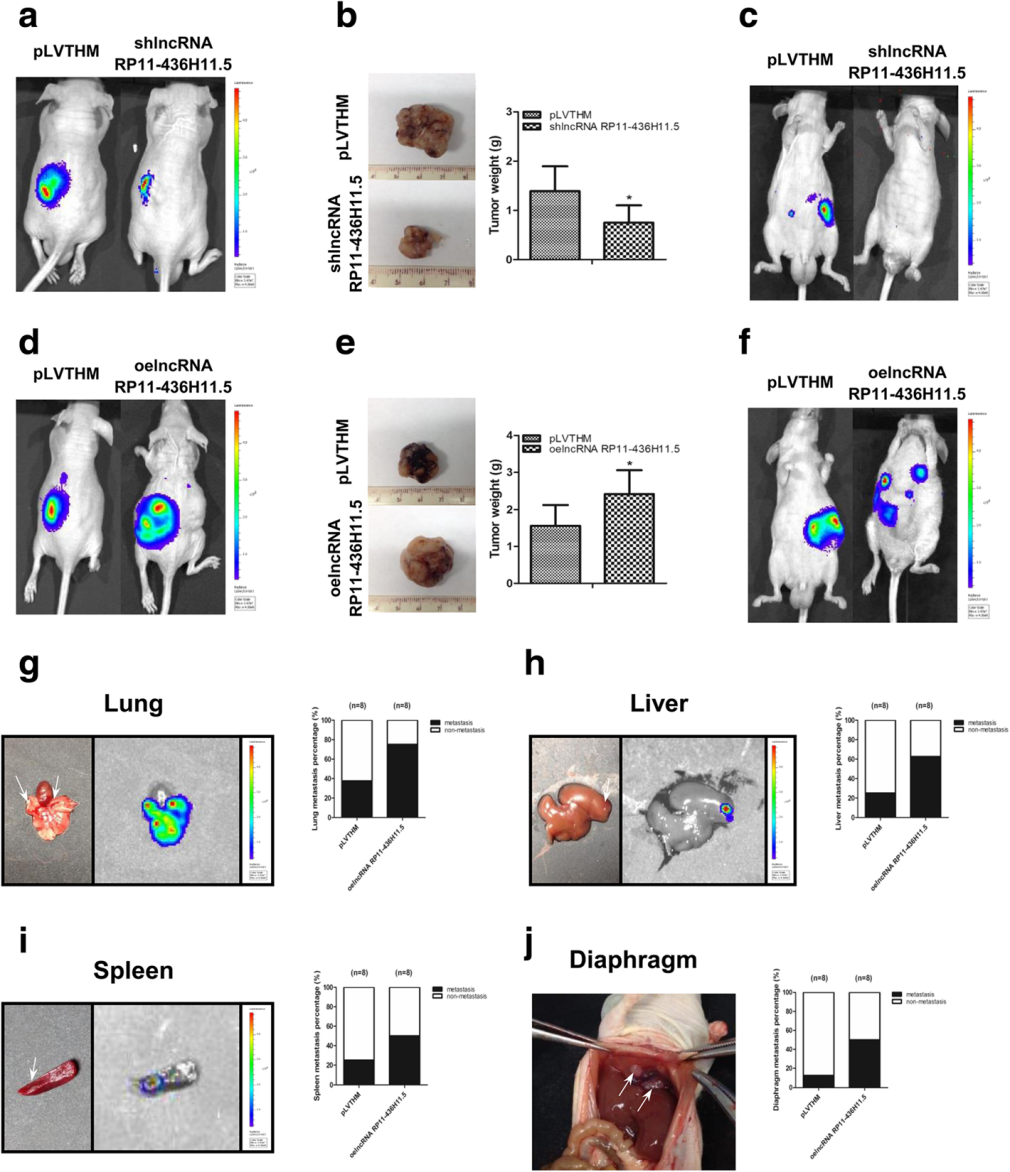
Deregulation of lncRNA RP11-436H11.5 suppresses RCC cell proliferation and invasion in RCC orthotopic xenografts. **a, b** and **c.** Representative IVIS images of tumor size (**a**), macroscopic appearance (**b**) and metastasis (**c**) in shlncRNA RP11-436H11.5 group vs control group. **d, e** and **f.** Representative IVIS images of tumor size (**d**), macroscopic appearance (**e**) and metastasis (**f**) in oelncRNA RP11-436H11.5 group vs control group. **g, h** and **i.** Representative macroscopic appearance and IVIS image of metastatic foci (white arrows) in lung (**g**), liver (**h**) and spleen (**i**). **j.** Representative macroscopic appearances of metastatic foci (white arrows) in diaphragm. **b** and **e.** Each sample was run in triplicate and in multiple experiments for mean ± SEM. *P < 0.05 compared to controls

A498 cells were labeled with luciferase expression and transfected with lncRNA RP11-436H11.5-shRNA and functional lncRNA RP11-436H11.5-cDNA. Then, the transfected cells were inoculated into the left renal capsule of nude mice. Tumor size and metastasis were monitored through an In Vivo Imaging System (IVIS). After 6 weeks, IVIS results revealed that a reduction of tumor luciferase expression was found in the shlncRNA RP11-436H11.5 group compared to the shRNA-control group (Fig. 6a). Meanwhile, the results also showed that deregulation of lncRNA RP11-436H11.5 effectively suppressed tumor size and metastasis (Fig. 6b and c). Conversely, oelncRNA RP11-436H11.5 increased tumor size and metastasis compared to the control group (Fig. 6d–f). Furthermore, oelncRNA RP11-436H11.5 promoted lung, liver, spleen and diaphragm metastasis (Fig. 6g–j).

Together, the results from Fig. 6a-j demonstrated that shlncRNA RP11-436H11.5 functioned as a tumor suppressor by inhibiting tumorigenesis and metastasis in RCC cells.

## Discussion

RCC is a common tumor-related cause of death worldwide. The incidence of RCC has increased from 61,560 to 63,990 over the past three consecutive years in the United States [1, 21, 22]. LncRNAs have been found to take part in many biological processes, that maintain relatively low expression levels and are primate specific [23]. Recent studies have shown that abnormal expression of lncRNAs can lead to tumor onset and progression [24–26]. Therefore, it is important to find new lncRNAs for the prevention and treatment of RCC.

Our previous study found that miR-335 suppressed RCC cell proliferation and invasion by repressing BCL-W expression [19]. However, the functional impacts of lncRNAs in miR-335-BCL-W-mediated tumorigenesis remain unclear. Based on our previous study, candidate lncRNAs were selected by a human lncRNA target prediction tool (DIANA TOOLS). The qRT-PCR screening results showed that lncRNA RP11-436H11.5 was upregulated in RCC tissues compared to adjacent normal renal tissues. In addition, RCC patients in the high lncRNA RP11-436H11.5 group showed a worse prognosis by Kaplan-Meier survival curves than those in the low lncRNA RP11-436H11.5.

Notably, recent studies have shown that lncRNAs can function as a miRNA sponge to regulate mRNA expression levels [27–29]. LncRNAs can sponge miRNAs by MREs to protect downstream mRNAs from repression [30]. For example, lncRNA BC032469 could upregulate hTERT expression to promote proliferation in gastric cancer by sponging miR-1207-5p [31]. LncRNA-BGL3 functioned as a ceRNA to cross-regulate the expression of PTEN by sponging 6 miRNAs [32]. Our previous study demonstrated that miR-335 suppressed RCC cell proliferation and invasion by repressing BCL-W. Subsequently, we considered whether lncRNA RP11-436H11.5 regulated miR-335-BCL-W-mediated RCC cell proliferation and invasion by functioning as a miRNA sponge. To prove this notion, luciferase reporter assays were applied to verify the binding effect of predicted MREs on the full-length lncRNA RP11-436H11.5 transcript. As our expected, miR-335-5p repressed the lncRNA RP11-436H11.5 reporter gene by complementary bindings. In addition, RNA-pull down assays further confirmed that lncRNA RP11-436H11.5 sponged miR-335-5p to derepress BCL-W expression. Furthermore, luciferase reporter assays revealed that ectopic overexpression of lncRNA RP11-436H11.5 increased BCL-W expression by sequestrating miR-335-5p. Taken together, we put forward for the first time that lncRNA RP11-436H11.5 functioned as a ceRNA by upregulating BCL-W expression by sponging miR-335-5p and promoted proliferation and invasion in RCC cells. Furthermore, deregulation of lncRNA RP11-436H11.5 expression led to depress BCL-W levels, hindering RCC cell growth.

From our study, we had reasons to believe that ectopic overexpression of lncRNA RP11-436H11.5 might be used as a biomarker of poor prognosis in RCC patients. Our study also revealed that lncRNA RP11-436H11.5 adjusted a miRNA/targeted gene transcript transformation to perform its functions in RCC pathogenesis. We considered that interruption of the lncRNA RP11-436H11.5-miR-335-BCL-W signals might help us find a new way to suppress RCC progression.

It is worth mentioning that the number of patients used for Kaplan-Meier analysis is low. However, these were all the tissues in our hospital during this period. More RCC tissues should be included in the study to yield convincing results. In addition, we all know that cross-regulation of ceRNAs at the gene level is exceedingly complicated. One lncRNA can regulate an army of miRNAs at the same time, and one miRNA can regulate multiple genes. Therefore, the miR-335-BCL-W signal may not be the only pathway targeted by lncRNA RP11-436H11.5 in RCC. On the other hand, there may be other lncRNAs that regulate the levels of key genes in RCC cell proliferation and invasion. Additional studies to find pivotal genes and related lncRNAs can help us better understand the pathogenesis and progression of RCC.

## Conclusions

In summary, lncRNA RP11-436H11.5 can function as a miR-335-5p decoy to derepress BCL-W and promote RCC cell proliferation and invasion. An orthotopic xenograft mouse model indicated that lncRNA RP11-436H11.5 could participate in miR-335-BCL-W-mediated RCC cell proliferation and invasion. LncRNA RP11-436H11.5 may be a novel therapeutic target and prognostic marker in RCC.

## Additional files

Additional file 1: Figure S1. A human lncRNA target prediction tool (DIANA TOOLS) was used to predict potential lncRNAs that could interact with miR-335-5p (PNG 8684 kb)

Additional file 2: Figure S2. A human miRNA target prediction tool (DIANA TOOLS) was used to find the predicted binding sites of miR-335-5p to lncRNA RP11-436H11.5 (PNG 6870 kb)

## Abbreviations

3′-UTR: 3′-untranslated region
CeRNA: Competing endogenous RNA
IVIS: In Vivo Imaging System
LncRNAs: Long non-coding RNAs
MiRNAs: microRNAs
MREs: miRNA response elements
NcRNAs: Non-coding RNAs
OS: Overall survival
RCC: Renal cell carcinoma
