# Supplementary figures and images for: LncRNA RP11-436H11.5, functioning as a competitive endogenous RNA, upregulates BCL-W expression by sponging miR-335-5p and promotes proliferation and invasion in renal cell carcinoma
Source: Mol Cancer. 2017 Oct 25;16:166. doi: 10.1186/s12943-017-0735-3 (PMC5657097; doi:10.1186/s12943-017-0735-3)

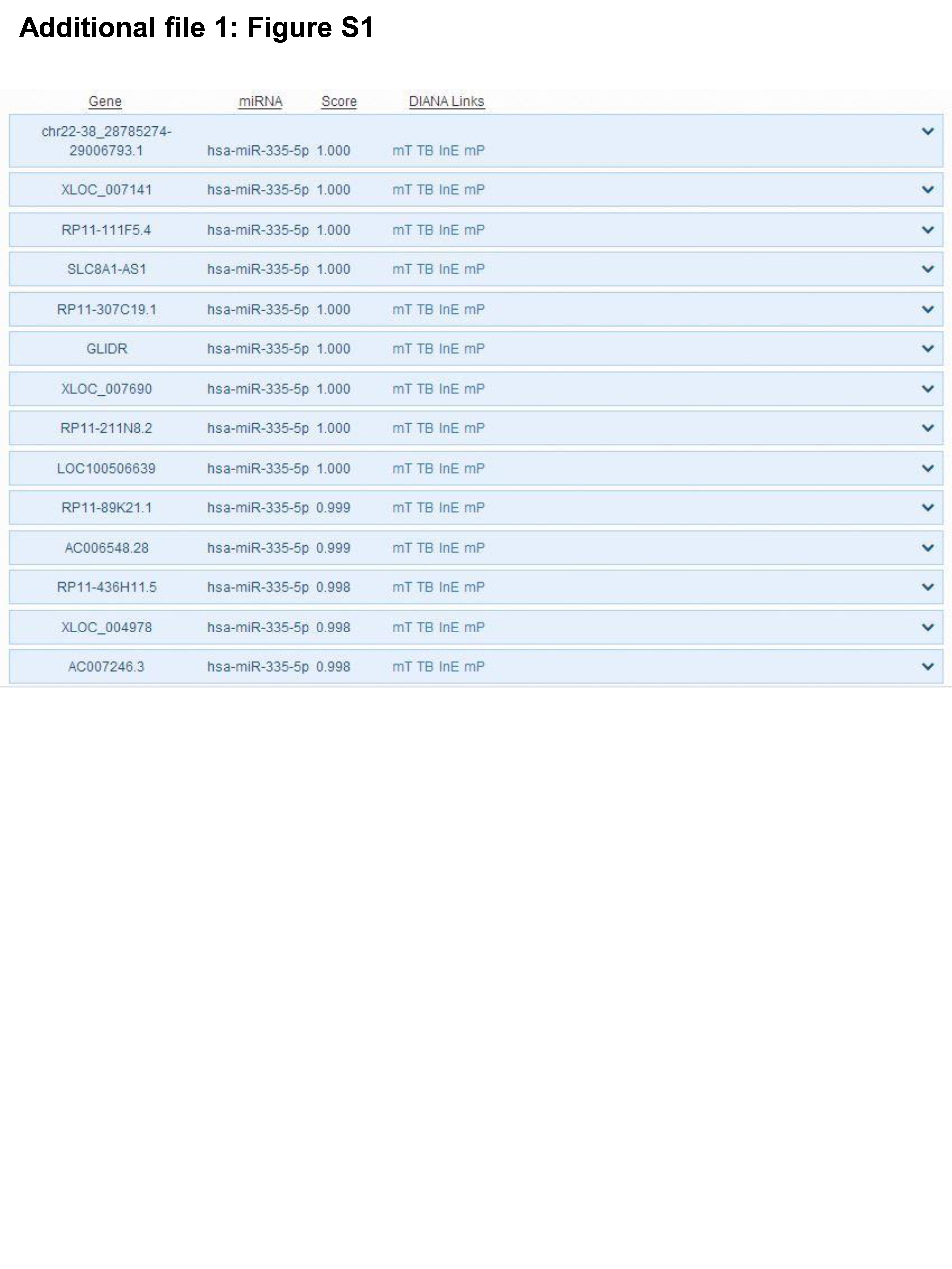

Supplement: Supplementary file 1 — A human lncRNA target prediction tool (DIANA TOOLS) was used to predict potential lncRNAs that could interact with miR-335-5p (PNG 8684 kb) [file 12943_2017_735_MOESM1_ESM.png]

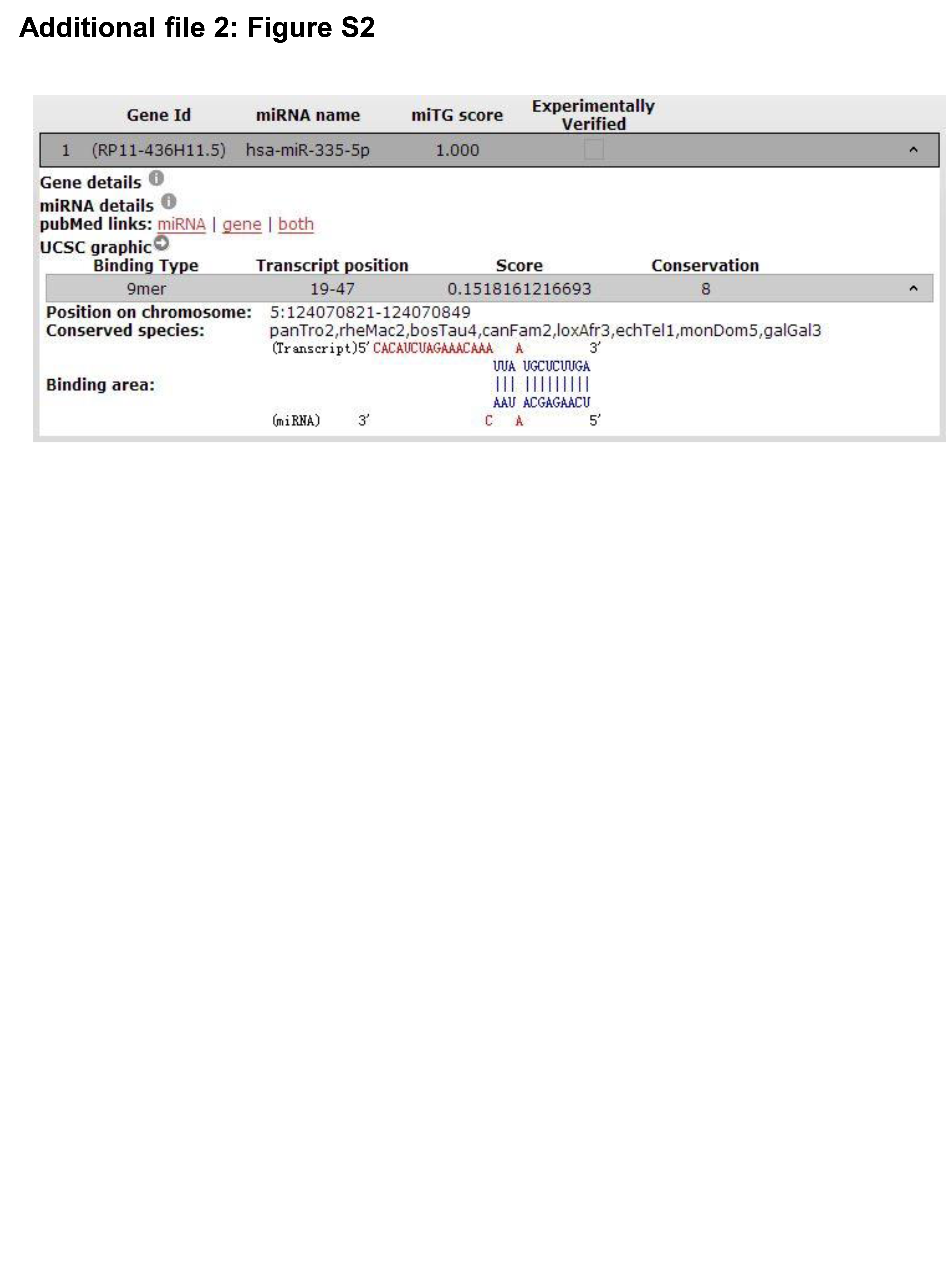

Supplement: Supplementary file 2 — A human miRNA target prediction tool (DIANA TOOLS) was used to find the predicted binding sites of miR-335-5p to lncRNA RP11-436H11.5 (PNG 6870 kb) [file 12943_2017_735_MOESM2_ESM.png]
